# Retraction: HBsAg Inhibits the Translocation of JTB into Mitochondria in HepG2 Cells and Potentially Plays a Role in HCC Progression

**DOI:** 10.1371/journal.pone.0292101

**Published:** 2023-09-21

**Authors:** The PLOS ONE Editors

Following the publication of this article [1], concerns were raised regarding multiple figures. Specifically,

- In the JTB Panel in Figure 1A, the following lanes appear to have similar patterns within the background:
   ⚬ Lanes 1 and 2;
   ⚬ Lanes 3 and 5.
- In the Bcl-XL Panel in Figure 2C, the following lanes appear to have similar patterns within the background:
   ⚬ Lanes 1 and 3;
   ⚬ Lanes 2 and 4.
- In the MMP-2 Panel in Figure 3E, lanes 3 and 6 appear to have similar patterns within the background.
- In the p-AKT panel in Figure 4D:
   ⚬ The bands in lanes 1 and 3 appear similar in addition to sharing similar patterns within the background;
   ⚬ Lanes 2 and 5 appear to have similar patterns within the background.

The corresponding and first authors provided files described as the original raw images underlying the panels in the above figures; however, the background patterns did not appear to match the published panels. The authors stated that they had applied background subtraction and changed the exposure, contrast, and color levels of the images using photo editing tools in order to improve the presentation of the published images. The authors stated that in Figure 1A, a lane was removed between lanes 4 and 5 when preparing the published panel. The *PLOS ONE* Editors remain concerned about these figures.

In light of the concerns affecting multiple figure panels that question the validity and reliability of these data, the *PLOS ONE* Editors retract this article.

BG and JSP did not agree with the retraction. YPL, XNY, AJ, THH, JJL, and JLR either did not respond directly or could not be reached.
